# Metals Coprecipitation with Barite: Nano-XRF Observation of Enhanced Strontium Incorporation

**DOI:** 10.1089/ees.2019.0447

**Published:** 2020-04-06

**Authors:** Heather A. Hunter, Florence T. Ling, Catherine A. Peters

**Affiliations:** Department of Civil & Environmental Engineering, Princeton University, Princeton, New Jersey.

**Keywords:** barite, coprecipitation, industrial wastewater, metals, solid solution, strontium, trace elements

## Abstract

Coprecipitation can be an effective treatment method for the removal of environmentally relevant metals from industrial wastewaters such as produced waters from the oil and gas industry. The precipitation of barite, BaSO_4_, through the addition of sulfate removes barium while coprecipitating strontium and other alkaline earth metals even when these are present at concentrations below their solubility limit. Among other analytical methods, X-ray fluorescence (XRF) nanospectroscopy at the Hard X-ray Nanoprobe (HXN) beamline at the National Synchrotron Light Source II (NSLS-II) was used to quantify Sr incorporation into barite. Thermodynamic modeling of (Ba,Sr)SO_4_ solid solutions was done using solid solution—aqueous solution (SS-AS) theory. The quantitative, high-resolution nano-XRF data show clearly that the Sr content in (Ba,Sr)SO_4_ solid solutions varies widely among particles and even within a single particle. We observed substantial Sr incorporation that is far larger than thermodynamic models predict, likely indicating the formation of metastable solid solutions. We also observed that increasing barite supersaturation of the aqueous phase led to increased Sr incorporation, as predicted by available kinetic models. These results suggest that coprecipitation offers significant potential for designing treatment systems for aqueous metals' removal in desired metastable compositions. Solution conditions may be optimized to enhance the incorporation of Sr by increasing sulfate addition such that the barite saturation index remains above ∼3 or by increasing the aqueous Sr to Ba ratio.

## Introduction

The recent expansion of hydraulic fracturing in gas-bearing shales has generated large volumes of flowback and produced water (FPW) containing high concentrations of the alkaline earth metals strontium, barium, and radium, as well as other metals and metalloids. These pose a risk to ecosystems and human health if these waste streams are released to the environment (Vidic *et al.*, 2013; Burton *et al.*, 2014; Jiang *et al.*, 2014; Vengosh *et al.*, 2014; Gregory and Mohan, 2015; Wang *et al.*, 2015, 2016; Lozano *et al.*, 2018; Toumari *et al.*, 2019).

Haluszczak *et al.* (2013) reported a median Ba concentration of 1,990 mg/L and a median Sr concentration of 2,330 mg/L from seven hydraulic fracturing wells in the Marcellus Formation. A median Ra concentration of 2,460 pCi/L was reported by Rowan *et al.* (2011) from a compilation of Marcellus Shale data. These concentrations far exceed the U.S. Environmental Protection Agency (U.S. EPA) maximum contaminant levels of 2 mg/L for Ba and 5 pCi/L for Ra as well as the nonenforceable health advisory level of 4 mg/L for Sr (U.S. EPA 2018b). Because of their toxicity and tendency to form scale that can clog pipes, wells, and downhole fractures (Hajirezaie *et al.*, 2017; Heberling *et al.*, 2017; Coll de Pasquali *et al.*, 2019), these metals must be removed before disposal or reuse (Mohammad-Pajooh *et al.*, 2018).

While treatment strategies such as ion exchange, osmosis, filtration, and electrodialysis can be effective for removing Sr, Ba, and Ra, chemical precipitation is the simplest method to implement and often the most cost effective (Ahmadun *et al.*, 2009; Fu and Wang, 2011; Zhang *et al.*, 2014; Bi *et al.*, 2016; U.S EPA, 2018a).

More broadly, coprecipitation is a simple, inexpensive, and effective method for the removal of a variety of radionuclides, metals, and metalloids from contaminated waters. In a coprecipitation reaction, similarities in charge, size, and crystal structure allow ions to substitute for one another in a newly forming solid. An important feature of coprecipitation reactions is that precipitation of trace elements can occur even when the aqueous solution is undersaturated with respect to the pure endmember containing that element. For instance, in Ra incorporation into barite, Ra^2+^ is often present at very low concentrations such that the solution is undersaturated with respect to RaSO_4_. However, if the solution is supersaturated with respect to barite, Ra can substitute for Ba and precipitate in a mineral that contains a mixture of both metals, that is, a solid solution (Rosenberg *et al.*, 2013).

Coprecipitation reactions are ubiquitous in nature and have been exploited by engineers and geochemists to remove contaminants from industrial wastewaters and contaminated groundwater (Myneni *et al.*, 1998; Hanor, 2000; He *et al.*, 2014; Prieto *et al.*, 2016; Drake *et al.*, 2018; Yuan *et al.*, 2018). For example, phosphate coprecipitation has been used to remediate soil and groundwater contaminated with lead, zinc, and cadmium (Conca and Wright, 2006; Flis *et al.*, 2011). Nuclear waste repository risk assessments consider coprecipitation of radionuclides with carbonates, sulfates, and clay minerals because they offer a more accurate prediction of radionuclide concentrations in migrating nuclear waste fluids (Curti, 1999; Bruno *et al.*, 2007; Curti *et al.*, 2010).

Sulfate coprecipitation has proven to be an effective treatment method for oil and gas FPW. In particular, barite (BaSO_4_) is an ideal host mineral for contaminant removal through coprecipitation because of its low solubility and ability to form solid solutions with a variety of cations and anions. The coprecipitation of the radionuclide Ra with barite has been extensively studied for removal of Ra and Ba from FPW (Kondash *et al.*, 2013; He *et al.*, 2014; Rosenberg *et al.*, 2014; Zhang *et al.*, 2014). Barite can also incorporate other harmful divalent cations, including cadmium, strontium, zinc, and lead (Zhu, 2004; Fernández-González *et al.*, 2013). Anions that can substitute for sulfate in barite include arsenate (HAsO_4_^2−^), chromate (CrO_4_^2−^), and selenate (SeO_4_^2−^) (Zhu, 2004; Tokunaga and Takahashi, 2017; Ling *et al.*, 2018). Barite's high density (4.48 g/cm^3^) is also advantageous for rapid settling of solid particles.

This article focuses on Sr coprecipitation in barite. Sr and Ba occur together naturally and are known to commonly coprecipitate as sulfates (Prieto *et al.*, 1997; L'Heureux and Jamtveit, 2002). Barite is much less soluble (with a solubility product, K_sp_, of 10^−9.98^) (Blount, 1977) than the strontium sulfate endmember, celestine (SrSO_4_) (K_sp_ = 10^−6.47^) (Reardon and Armstrong, 1987). As with the (Ba, Ra)SO_4_ solid solution, Sr incorporation in barite can occur even when the aqueous solution is undersaturated with respect to celestine. However, unlike Ra, the strong thermodynamic preference for Ba incorporation makes removal of Sr challenging. For example, He *et al.* (2014) investigated the removal of Ba and Sr from Marcellus Shale flowback water through sulfate precipitation and found that Ba removal was extensive while Sr removal was significantly lower.

This article includes a theoretical analysis of models that can be used to predict the potential for contaminant removal from waste streams. An important first-level predictor is the degree of supersaturation of each endmember. Supersaturation is quantified by the saturation index, *SI*, a measure of how far the solution is from equilibrium with the solid phase. For the (Ba,Sr)SO_4_ system, the two endmember saturation indices are:

(1) $$SI_{BaSO_{4}}=log\left(\frac{\left\{Ba^{2+}\right\}\left\{S{O_{4}}^{2-}\right\}}{K_{sp,BaSO_{4}}}\right).$$

(2) $$SI_{SrSO_{4}}=log\left(\frac{\left\{Sr^{2+}\right\}\left\{S{O_{4}}^{2-}\right\}}{K_{sp,SrSO_{4}}}\right).$$

Values of *SI* greater than zero indicate supersaturation and the tendency for precipitation. In a treatment system, supersaturation may be achieved through sulfate dosing using, for example, Na_2_SO_4_. Conventional engineering models of coprecipitation use what we call the endmember solubility approach. This approach accounts for the formation of only the two pure endmembers while neglecting the possibility of solid solutions. This article begins with an overview of thermodynamic models that describe equilibrium between solid solution and aqueous solution (SS-AS) systems (Glynn, 2000; Prieto, 2009).

The experimental study was designed to investigate Sr incorporation into barite precipitates for different supersaturation conditions. The initial concentrations were selected to test the possibility of using barite coprecipitation to remove Sr from wastewaters that would not favor the formation of pure SrSO_4_.

We used nanoscale-resolution X-ray fluorescence (XRF) spectroscopy, termed “nano-XRF,” conducted at the Hard X-ray Nanoprobe (HXN) beamline at the National Synchrotron Light Source II (NSLS-II). The elemental fluorescence maps were used to quantify Sr incorporation within individual micron-sized barite particles. The unique, high-resolution data provide direct evidence of the formation of solid solutions in barite precipitated homogeneously from solution. The nano-XRF data enable observation of variability in Sr incorporation with solution conditions as well as particle-to-particle variability within a single experiment. These observations can also provide a record of particle growth history and thereby give information on how changes in solution conditions effect solid solution formation.

Larger batches of solid precipitate in each experiment were also prepared and analyzed using bulk XRF. Postprecipitation aqueous-phase compositions were also determined. Finally, in addition to comparing the Sr incorporation results to thermodynamic predictions, the results are also discussed in the context of kinetic processes that may limit the applicability of SS-AS theory.

## SS-AS Thermodynamic Theory

Until now, SS-AS theory has been investigated primarily in the context of geochemical mineral systems. We discuss this theory in the context of its relevance for engineered systems in which prediction of trace element incorporation into solid solutions would enable achievement of wastewater treatment goals. The theory is also used as a comparative reference for inferences drawn from the experimental observations.

Thermodynamic equilibrium of a binary solid-solution B_1-x_C_x_A in equilibrium with an aqueous solution begins with two mass action equations describing the solubility of the endmembers BA and CA:

(3) $$\left\{B^{+}\right\}\left\{A^{-}\right\}=K_{BA}X_{BA}\gamma_{BA}.$$

(4) $$\left\{C^{+}\right\}\left\{A^{-}\right\}=K_{CA}X_{CA}\gamma_{CA}.$$

where $K_{BA}$ and $K_{CA}$ are the solubility products of pure endmembers BA and CA, and $\left\{B^{+}\right\}$, $\left\{C^{+}\right\}$, and $\left\{A^{-}\right\}$ are the ion activities in the aqueous phase. In the solid phase, $X_{BA}$ and $X_{CA}$ are the solid solution mole fractions of the endmembers, for example:

(5) $$X_{BA}=\frac{n_{BA}}{n_{BA}+n_{CA}}.$$

where $n_{BA}$ is the number of moles of component BA in the solid and $n_{CA}$ is the number of moles of component CA. The solid-phase activity coefficients are $\gamma_{BA}$ and $\gamma_{CA}$.

Researchers have proposed different manipulations of the mass action equations to determine possible equilibrium states of solid solutions. The most widely accepted of these is the model proposed by Lippmann and expanded by Glynn *et al.* (Lippmann, 1980; Glynn *et al.*, 1990) Lippmann introduced the total solubility product, ΣΠ:

(6) $$\Sigma \Pi =\left(\left\{B^{+}\right\}+\left\{C^{+}\right\}\right)\left\{A^{-}\right\}.$$

which represents the saturation state of a solid solution. At equilibrium with an aqueous phase, both mass action equations must be satisfied, so the equilibrium value of ΣΠ in terms of solid-phase composition is found by substituting Equations (3) and (4):

(7) $$\Sigma \Pi_{eq}=K_{BA}X_{BA}\gamma_{BA}+K_{CA}X_{CA}\gamma_{CA}.$$

Lippmann denoted this expression, the solidus, as it describes the solid-phase compositions that satisfy the requirements of equilibrium. By algebraic rearrangement, equilibrium is also expressed in terms of solution phase activity fractions.

(8) $$\Sigma \Pi_{eq}={\left(\frac{X_{B,aq}}{K_{BA}\gamma_{BA}}+\frac{X_{C,aq}}{K_{CA}\gamma_{CA}}\right)}^{-1}.$$

where the cation activity fractions are given by:

(9) $$X_{B,aq}=\frac{\left\{B^{+}\right\}}{\left\{B^{+}\right\}+\left\{C^{+}\right\}}.$$

and

(10) $$X_{C,aq}=\frac{\left\{C^{+}\right\}}{\left\{B^{+}\right\}+\left\{C^{+}\right\}}.$$

Lippmann termed this expression the solutus. The solidus and solutus plotted together on a binary phase equilibrium diagram is referred to as a Lippmann diagram.

**FIG. 1.**
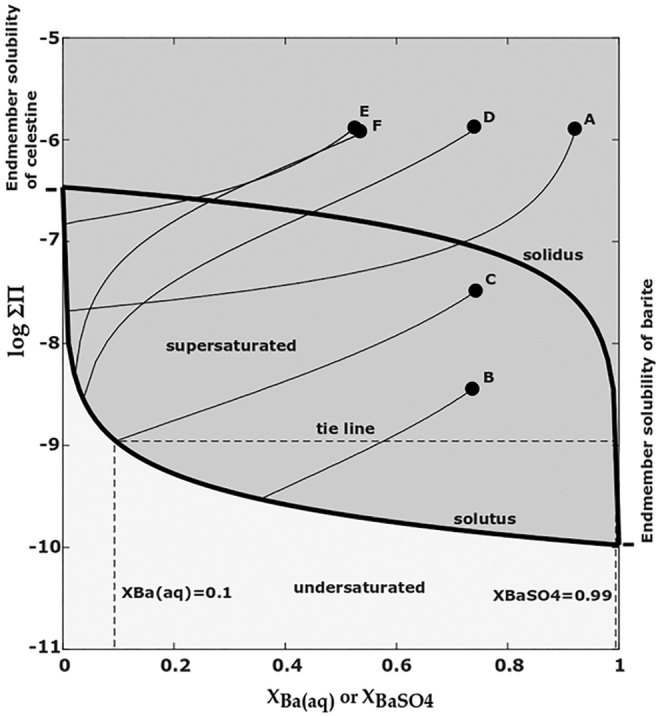
Lippmann diagram representing possible equilibrium states for the (Ba,Sr)SO_4_ solid solution/aqueous solution system with example reaction paths for supersaturated solutions.

For the (Ba,Sr)SO_4_ SS-AS system, the Lippman diagram is shown in Fig. 1 in which the solid-phase activity coefficients have been assumed to be unity. Under this assumption, the only parameters required are the solubility products of the two endmembers. The solubilities of pure barite and pure celestine are represented as the values of $\Sigma \Pi atthefarright\left(X_{BaSO_{4}}=1\right)$ and far left $\left(X_{BaSO_{4}}=0\right)$, respectively, of the diagram.

The solid solution composition corresponding to an equilibrium aqueous composition is determined by a horizontal tie line connecting the solutus to the solidus. For example, as indicated on Fig. 1, an aqueous solution with $X_{Ba,aq}$ equal to 0.1 corresponds to a solid-phase composition with $X_{BaSO_{4}}$of 0.99. This demonstrates an important feature of the (Ba,Sr)SO_4_ system as predicted by SS-AS theory. Even though Sr can be removed from solution below the solubility limit of SrSO_4_, the almost three order of magnitude difference between the solubilities of barite and celestine means that a wide range of aqueous compositions are in equilibrium with almost pure barite. That is, SS-AS theory predicts that only systems with a high ratio of Sr^2+^ to Ba^2+^ will have appreciable Sr in the solid phase, suggesting that it is challenging to remove the more soluble ion (Sr^2+^) from solution. The ideality of the (Ba,Sr)SO_4_ solid solution has been the subject of debate, in part, due to the difficulty of separating thermodynamic effects from kinetic effects when studying solid solutions (Prieto *et al.*, 2000; Heberling *et al.*, 2017; Weber *et al.*, 2018). Considering the solid solution as nonideal will raise the position of the solutus compared with an ideal solid solution, or in other words, increase the solubility of the solid solution (Glynn, 2000). The position of the solidus will also shift up for a nonideal solid and, in general, less trace element incorporation into the solid will be predicted. While some researchers have argued that (Ba,Sr)SO_4_ solid solutions can be considered as ideal, more recent work by Prieto *et al.* (2000), using theoretical calculations of thermodynamic mixing properties, shows significant nonideality. These authors predict a wide miscibility gap over much of the solid solution composition range. The fact that solid solutions of (Ba,Sr)SO_4_ have been observed over the entire composition range is explained as the formation of metastable solid solutions. The values of the interaction parameters used in the nonideal mixing model are still uncertain and an active area of research (Heberling *et al.*, 2017).

Nonequilibrium conditions are plotted on a Lippmann diagram to represent how far from equilibrium an aqueous solution is. Points plotting above the solutus represent a supersaturated solution. While a Lippmann diagram can show that a solution is supersaturated, it is not sufficient to predict which solid-phase composition is expected to form. Glynn *et al.* (1990) introduced a solution method by introducing equations for conservation of mass of the two cations and conservation of charge in the solid (Supplementary Data). The resulting system of equations is solved with an iterative procedure that includes an independent calculation of the aqueous-phase activity coefficients, as well as an ion association model to calculate the speciation factors. Nonideal solid solutions could also be considered and would require a separate calculation of the solid-phase activity coefficients for any given solid composition.

After solving for the equilibrium solid and aqueous-phase compositions, the theoretical reaction path showing the evolution of the aqueous-phase composition is calculated [Eqs. (S8)–(S10) in Supplementary Data]. In this work, we have assumed the precipitate to have a homogenous composition; that is, at every point along the reaction path the solid forms with its final equilibrium composition (Berthelot/Nernst type precipitation). Example reaction paths are plotted in Fig. 1. The initial solution conditions for these paths are included in the Supplementary Table S1. The first important observation is that all reaction paths move toward the left, indicating enrichment of the more soluble ion, Sr^2+^, in the aqueous phase. Second, the higher the initial ΣΠ, the further to the left are the resulting endpoints on the solutus curve (paths B, C, and D). The resulting tie line point on the solidus also moves farther to the left indicating increased incorporation of Sr. This illustrates that larger sulfate addition can produce a solid phase with higher Sr molar fraction. Third, a solution with a higher $X_{Ba,aq}$ can end up with a lower final $X_{BaSO_{4}}$ than a solution with a lower initial $X_{Ba,aq}$ (paths A and D, respectively) showing that a larger ratio of aqueous Sr^2+^ to Ba^2+^ does not necessarily mean a larger fraction of Sr in the solid. Lastly, coincident points in the supersaturation region can result in very different equilibrium compositions. For example, while points E and F are similar in both initial ΣΠ and $X_{Ba,aq}$, the systems take very different paths due to stoichiometric constraints not visible on a Lippmann diagram. System F initially has equimolar Ba^2+^, Sr^2+^, and SO_4_^2−^, whereas system E initially has four times as much SO_4_^2−^ as Ba^2+^ and Sr^2+^ (with charge balance provided by Na^+^ and Cl^−^ in both cases). In system F, almost all the sulfate reacts with Ba and Ba is removed from solution, resulting in a low final position on the solutus line. In system E, which has excess sulfate, the Ba is again removed, but much of the sulfate remains in solution and the system has a high final position on the solutus line. The sulfate limitation would cause system F to produce a solid with only $X_{SrSO_{4}}$ of 0.024, whereas system E is expected to form solids with $X_{SrSO_{4}}$ of 0.43. This example demonstrates that it may be possible to increase trace element incorporation solely by adjusting the cation to anion ratio.

In this study, we chose conditions to explore a region of the Lippmann diagram that would highlight the differences between SS-AS theory and the simpler endmember solubility model. We chose two barite supersaturation cases such that the low barite *SI* experiment was supersaturated with respect to BaSO_4_ and undersaturated with respect to SrSO_4_, and the high barite *SI* experiment was supersaturated with respect to both endmembers. These conditions are interesting in that the undersaturation of SrSO_4_ in the low *SI* case and the very moderate supersaturation of SrSO_4_ in the high *SI* case would lead to an endmember solubility model prediction of no Sr removal in both cases. Even though SrSO_4_ is supersaturated in the high *SI* experiment, BaSO_4_ is much more supersaturated, so that an endmember solubility model would predict all available sulfate is reacted with Ba. Further details on these predictions are included in the Supplementary Data.

The two *SI* conditions also allowed us to examine the effect of a higher barite *SI* in the context of available kinetic models for solid solution precipitation and Sr incorporation. It has long been known that degree of supersaturation can substantially increase precipitation rate. For example, He *et al.* (1995) found that the time required for the onset of barite nucleation decreased from ∼30 s to ∼1 s when the saturation index was increased from 3 to 4.

Furthermore, solid precipitation rates can have important effects on the incorporation of trace elements. For example, Rosenberg *et al.* (2014) found that the amount of Ra incorporation into barite varied by a factor of two depending on precipitation kinetics, which they related to the degree of barite supersaturation. Likewise, Weber *et al.* (2018) and Deng *et al.* (2019) observed higher levels of Sr incorporation into barite than predicted by thermodynamics and suggested that kinetic factors caused the discrepancy. In geological systems, when precipitation is fast, solids can form with nonequilibrium compositions and are effectively frozen in a metastable state due to slow ion diffusion in the solid phase (Watson, 2004). Nonequilibrium effects will also likely be important in engineered precipitation treatment systems because precipitation is typically employed when concentrations are high and rapid precipitation can be achieved.

The work of Thien *et al.* (2014), Pina and Putnis (2002), and Noguera *et al.* (2010), has sought to model the nucleation and growth kinetics of coprecipitation reactions to capture these nonequilibrium effects. Thien *et al.* developed a kinetic model of trace element incorporation for moderately supersaturated solutions where growth dominates over nucleation and were able to match experimentally observed decreases in Cd incorporation into calcite with increasing growth rate.

The work of Pina and Putnis focuses on trace element partitioning during nucleation rather than growth and suggests that Sr incorporation into barite should increase with increasing *SI* of barite (Pina and Putnis, 2002; Prieto *et al.*, 2016). This is because, in classical nucleation theory, the nucleation rate is strongly dependent on the interfacial tension of a substance, and interfacial tension is in turn related to solubility. The more soluble compound, in this case SrSO_4_, has a lower interfacial tension and hence at higher barite saturation (higher nucleation rate) the portion of the solid consisting of SrSO_4_ should increase.

The work of Noguera *et al.* models trace element incorporation during both nucleation and growth and yields sizes and compositions of individual particles as a function of time. The authors used their model to demonstrate that the composition of coprecipitated particles can be very sensitive to initial solution conditions and suggest that this feature might be used to “engineer the particle characteristics into a chosen state.” Thus, designing an engineered treatment system to produce a desirable metastable composition is a potential strategy to maximize contaminant removal and kinetic models can offer insight into which metastable composition will form.

## Experimental Methods

Initial solution concentrations of Ba^2+^, Sr^2+^, and SO_4_^2−^ were selected to investigate the effects of two conditions of barite supersaturation (summarized in Table 1). The Ba^2+^ and SO_4_^2−^ concentrations were selected to be equimolar. The Sr^2+^ to Ba^2+^ molar ratio was 1:3 in the high SI experiment and 1:2.3 in the low SI experiment. Aqueous-phase activity coefficients were calculated using the Pitzer formulation in PHREEQC and were found to be 0.8 for all three solutes in the low SI condition and 0.6 in the high SI condition.

**Table 1.** Initial Solution Compositions and Final Aqueous-Phase Cation Concentrations: Observed by Experiments and Predicted by SS-AS Thermodynamic Theory

	Low Barite Supersaturation	High Barite Supersaturation
Initial conditions		
[SO_4_^2−^] (mM)	0.214	1.5
[Sr^2+^] (mM)	0.095	0.5
[Ba^2+^] (mM)	0.214	1.5
Barite *SI*	2.34	3.80
Celestite *SI*	−1.20	0.11
ΣΠ	10^−7.3^	10^−5.9^
Final conditions observed		
[Sr^2+^] (mM)	$\left(4.32\pm 0.91\right)\times 10^{-2}$	$\left(2.22\pm 0.25\right)\times 10^{-1}$
Sr^2+^ % removal	55±9.6%	56±4.9%
[Ba^2+^] (mM)	$\left(1.52\pm 0.25\right)\times 10^{-2}$	$\left(1.75\pm 0.18\right)\times 10^{-1}$
Ba^2+^ % removal	93±1.2%	88±1.2%
Final conditions predicted by SS-AS theory		
[Sr^2+^] (mM)	$9.45\times 10^{-2}$	$4.89\times 10^{-1}$
Sr^2+^ % removal	1%	2%
[Ba^2+^] (mM)	$1.18\times 10^{-2}$	$2.02\times 10^{-2}$
Ba^2+^ % removal	94%	99%

Experiments were conducted by first preparing stock solutions using purchased salts of BaCl_2_ (Fisher), SrCl_2_ (Acros), and Na_2_SO_4_ (Fisher). Solutions of BaCl_2_ and SrCl_2_ were added to a Na_2_SO_4_ solution in a disposable cuvette and shaken vigorously. The cuvette was placed in an ultraviolet spectrometer and the absorbance at an incident light wavelength of 450 nm was recorded to monitor the formation, growth, and settling of particles. Samples were drawn from the aqueous suspension during the phase of particle settling, which was 0.5–2 h after initial mixing. Samples for aqueous phase analysis were collected concurrently, filtered through a 0.2 μm PTFE membrane filter (Fisherbrand), and diluted with a 70% HNO_3_ solution. Ba^2+^ and Sr^2+^ concentrations were then measured using a Thermo Neptune inductively coupled plasma mass spectrometer (ICP-MS).

The solids in the aqueous suspension samples were prepared for nano-XRF as follows: a 1–3 μL drop of each sample was deposited on a cantilevered silicon wafer (Norcada) and allowed to dry. SEM imaging (high-vacuum mode, 5 keV, Quanta 200 FEG Environmental-SEM) was done to locate particles of interest for nano-XRF analysis, and to visualize the shapes and sizes of particles. Particles were “of interest” based primarily on morphology; there was no intent to randomly select particles for representativeness.

The synchrotron-based nano-XRF imaging is similar to our work for arsenic coprecipitation in barite particles (Ling *et al.*, 2018). Individual particles, sized 10 microns or less, were raster scanned with incident energy of 16.1 keV. XRF spectra were collected with resolution as low as 30 nm. Relative moles of Ba and Sr were quantified from photon counts by developing calibration curves of known fluorescence intensities for specific (Ba,Sr)SO_4_ solid solutions. These were used to generate nano-XRF maps of $X_{SrSO_{4}}$. Further details of the nano-XRF imaging and the procedure for quantifying mole fractions are provided in the Supplementary Data.

Bulk XRF measurements were performed for solids from large batch experiments, in which solutions were mixed and left overnight. Solids were collected, dried, and ground. Two-inch-diameter circular pellets were formed by mixing with a cellulose binder (SPEX Ultrabind 3644) and compressing at 4 metric tons for 2 min. XRF analysis was done on a Rigaku Supermini 200 with a 50 kV X-ray tube. Elemental mass percentages were calculated with the ZSX Primus (Rigaku) software package. All elemental percentages, except Ba, Sr, and S, were found to be negligible, so stoichiometry assuming Sr substitution for Ba was used to calculate $X_{SrSO_{4}}$.

## Results & Discussion

### Nano-XRF and bulk XRF results

**FIG. 2.**
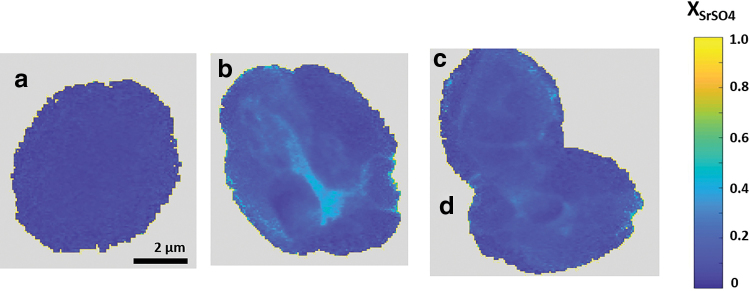
Nano-XRF maps of Sr content in particles a–d precipitated under high saturation index (barite *SI* = 3.80) conditions. Color map indicates celestine mole fraction.

**FIG. 3.**
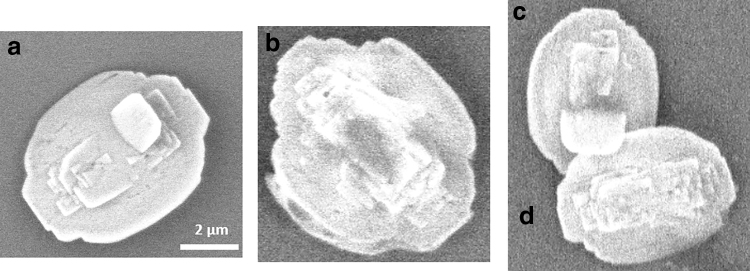
SEM images of particles precipitated under high *SI* conditions. Letters correspond to particles labeled in Figure 2.

Table 1 summarizes the observed final aqueous-phase concentrations and Table 2 summarizes the observed Sr incorporation into solid precipitates. For the high barite *SI* experiment, nano-XRF particle maps of $X_{SrSO_{4}}$ are presented in Fig. 2, and corresponding SEM images are in Fig. 3. The four particles imaged have consistent morphologies and are ∼5 μm in size. They exhibit relatively homogenous Sr incorporation, with a range of $X_{SrSO_{4}}$ values that spans the average value measured from bulk XRF. For each particle, this value is the average of 25 pixels near the center. Particle b shows a small region of higher Sr incorporation with $X_{SrSO_{4}}$ of 0.30 to 0.40.

**Table 2.** Experimental Observations of SrSO_4_ Mole Fractions in Solid Precipitates in Comparison to Predictions from SS-AS Thermodynamic Model, Conventional Engineering Model, and Kinetic Models

		$X_{SrSO_{4}}$	
		Low Barite Supersaturation	High Barite Supersaturation
Experimental observations	Bulk XRF	0.048	0.105
	Nano-XRF range	0.061 to 0.909	0.051 to 0.109
Thermodynamic model predictions	Conventional engineering model	0	0
	SS-AS thermodynamic model	0.0025	0.0074
Kinetic model predictions with literature parameters^a^	$\sigma_{BaSO_{4}}=$ 125 mJ/m^2^ and $\sigma_{SrSO_{4}}=$ 97 mJ/m^2^	0.0030	0.0120
Kinetic model predictions with adjusted parameters	$\sigma_{BaSO_{4}}=$ 150 mJ/m^2^ and $\sigma_{SrSO_{4}}=$ 95 mJ/m^2^	0.0100	0.0831

^a^ Parameters are from Pina and Putnis (2002).

**FIG. 4.**
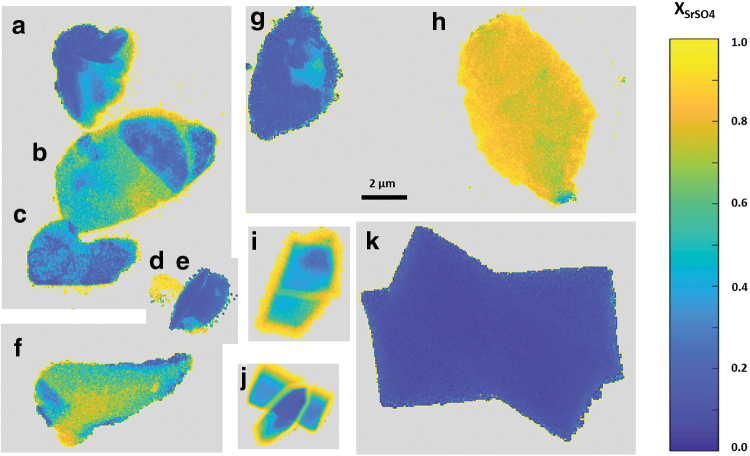
Nano-XRF maps of Sr content in particles a–k precipitated under low saturation index (barite *SI* = 2.34) conditions. Color map indicates celestine mole fraction.

**FIG. 5.**
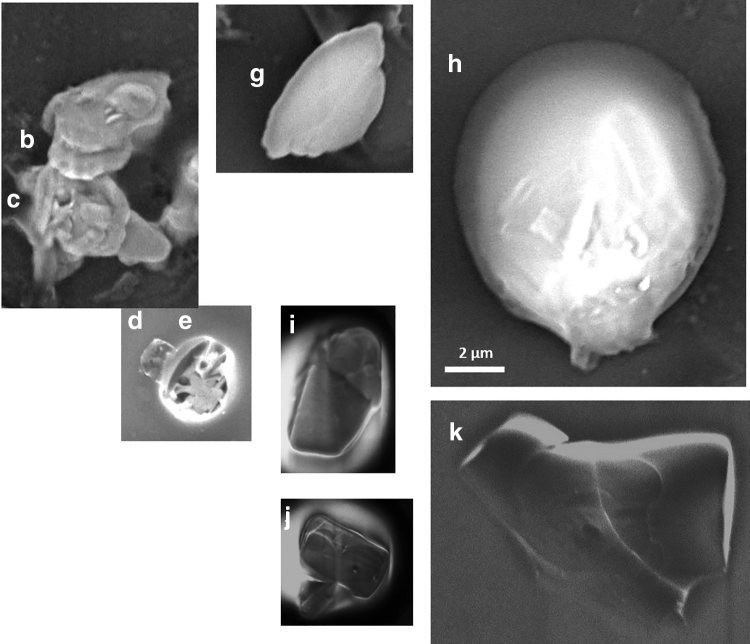
SEM images of selected particles precipitated under low *SI* conditions. Letters correspond to particles labeled in Figure 4. Particles a and f could not be located in the SEM.

For the low barite *SI* experiment, nano-XRF particle maps are presented in Fig. 4, and corresponding SEM images are in Fig. 5. (Some particles moved during transport and we were unable to locate particles a and f in the SEM.) The particles in this experiment show much wider variety in size, shape, and Sr content than those precipitated at high *SI*. Particle k has Sr incorporation similar to the bulk XRF value. Particle d approximated pure SrSO_4_. Several particles exhibited compositional zonation where the amount of Sr incorporation varies spatially within the particle, a phenomenon that we have previously reported for arsenic incorporation in barite (Ling *et al.*, 2018). Particles i and j are examples of this with a barite-rich core and a celestine-rich rim with $X_{SrSO_{4}}$ as high as 0.87. The high values of $X_{SrSO_{4}}$ observed in this experiment suggest that barite has significant capacity for Sr incorporation and that it is possible to form a complete series of Ba_x_Sr_1-x_SO_4_ solid solutions during precipitation.

### Comparison to SS-AS thermodynamic predictions

**FIG. 6.**
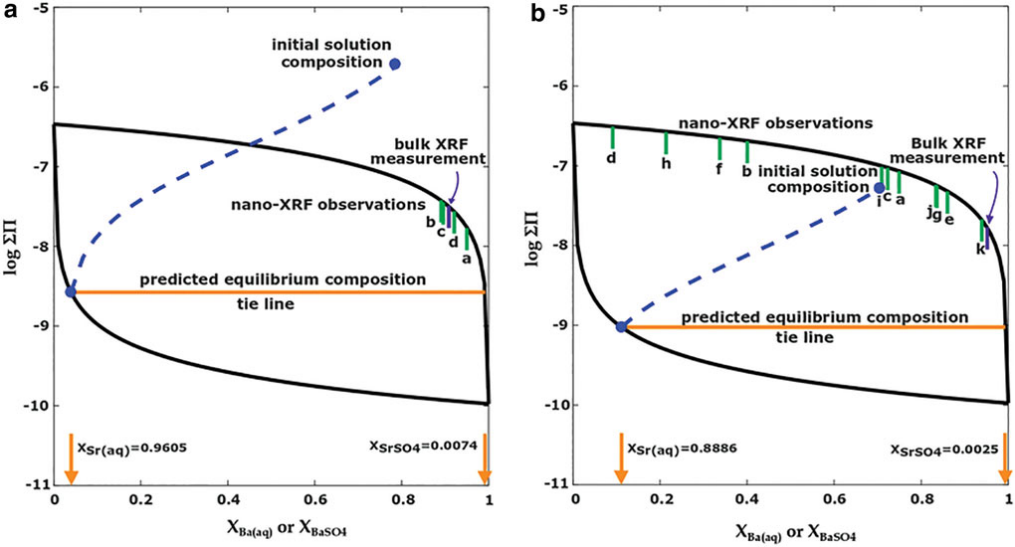
Lippmann diagrams for (Ba,Sr)SO_4_ solid solutions, showing experimental observations of solid-phase compositions and SS-AS thermodynamic model predictions. **(a)** High barite *SI* experiment. Letters correspond to particles identified in Figure 2. **(b)** Low barite *SI* experiment. Letters correspond to particles identified in Figure 4.

The nano-XRF observations and bulk XRF measurements of solid-phase incorporation of Sr are shown on the Lippmann diagrams in Fig. 6. For nano-XRF values, the letters correspond to the particles labeled in Figs. 2 and 4. Also shown are the reaction paths and equilibrium tie lines predicted from SS-AS theory using the Glynn method with Berthelot/Nernst type precipitation for the initial ΣΠ values corresponding to the experimental initial solution conditions.

For both experiments, SS-AS thermodynamic theory predicts that the solids will consist of <1% celestine (Table 2), which is substantially less than what was observed. The low *SI* experiment had 19 times more Sr incorporation than predicted. The high *SI* experiment had 14 times more Sr incorporation. Even if we had used the SS-AS model with solid-phase activity coefficients other than unity, it still would not have come close to predicting the large Sr incorporation observed. While the experimental values did not match SS-AS thermodynamic predictions, the experimental observation of high Sr incorporation at higher barite SI is, however, consistent with the trend predicted by the SS-AS theory. This can be visualized on a Lippmann diagram: for a given aqueous Ba:Sr ratio, as the initial ΣΠ (and barite *SI*) are increased, the system moves further to the left which corresponds to a higher equilibrium $X_{SrSO_{4}}$.

The extent of Sr incorporation was found to be higher in the high *SI* experiment than in the low *SI* experiment. As stated earlier, increasing *SI* is known to increase the nucleation and growth rates of precipitation, so that the effect of kinetics on precipitation becomes more dominant as *SI* increases (Rosenberg *et al.*, 2014; Prieto *et al.*, 2016). These observations are in agreement with previous work suggesting that precipitation of the more soluble endmember (SrSO_4_) will be kinetically favored over the less soluble endmember (BaSO_4_) when a solution is far from equilibrium (Pina and Putnis, 2002; Weber *et al.*, 2018). However, it contrasts with experimental work by Tokunaga *et al.* (2018). who found decreasing Sr incorporation with increasing *SI*.

We propose that kinetic effects may also explain why there was more varied Sr incorporation in the low *SI* experiment. Slower nucleation and growth rates in the lower *SI* case would allow time for precipitation to respond to changing solution conditions. The first precipitating particles would experience higher Ba^2+^ activities and hence precipitate with a higher barite mole fraction than particles that form later. Accordingly, particles with high barite mole fractions, such as particles e, g, and k, likely formed earlier than particles with low barite mole fractions, such as particles d, f, and h. Particles with barite-rich cores and celestine-rich rims (particles i and j) may have first nucleated when solution conditions favored barite formation and were still growing when celestine precipitation became favorable.

### Comparison to kinetic models

Like previous studies (Thien *et al.*, 2014; Weber *et al.*, 2018; Deng *et al.*, 2019), our results suggest that thermodynamics alone are not sufficient to explain the observed Sr partitioning in (Ba,Sr)SO_4_ precipitates. To explore the possibility that kinetic processes of nucleation and growth affect the incorporation of Sr, we also compared our results with two available kinetic models.

First, the model by Pina and Putnis (2002) extends classical nucleation theory by considering nucleation rate to be a function of the solid composition. To put it another way, the solid composition with the highest nucleation rate is the most likely to form. Theoretically, the more soluble endmember (SrSO_4_) has a smaller interfacial tension, and therefore, has less of an energy barrier for nucleation. In that model, either increasing the saturation index of the solution or decreasing the interfacial tension of the solid will result in an increase in the nucleation rate. However, the effect of interfacial tension is stronger, so that the reduced nucleation energy barrier of SrSO_4_ is amplified as the nucleation rate increases. Thus, that model predicts that precipitation of the SrSO_4_ will be kinetically favored, and increasing barite *SI* will increase Sr incorporation.

While the experimental observations agree with the predicted trend that increasing barite *SI* will increase Sr incorporation; using our experimental conditions in that model leads to a prediction of less Sr incorporation than observed in our experiments. Using values of interfacial free energy, σ, and the pre-exponential factor, Γ, taken from Pina and Putnis, the model predicts a celestine mole fraction of 0.0101 for our high *SI* case and zero for our low *SI* case. These values are the predicted compositions of the first nuclei and therefore, should correspond to the centers of our particles. The nano-XRF observations revealed substantially higher Sr incorporation than these predictions.

These differences may, in part, be attributed to uncertainties in the values of the interfacial free energies of barite and celestine, to which the model is very sensitive. There is quite a wide range of values reported in the literature such as those in Table 2 (Sangwal, 1989; Wu and Nancollas, 1999). Adjustments of the values of $\sigma_{SrSO_{4}}$ and $\sigma_{BaSO_{4}}$ within the reported range (Table 2) resulted in predicted Sr content high enough to match the nano-XRF observations of *X*_SrSO4_ in our experiments.

We also compared our results with the binary SS-AS kinetic precipitation model developed by Noguera *et al.* (2010). Unlike the model of Pina and Putnis, Noguera's model also considers particle growth and tracks the entire particle population over time. Some of the particles from our low *SI* case showed compositional zonation with a barite-rich core and celestine-rich rim. This agrees qualitatively with Noguera's kinetic model.

### Implications for wastewater treatment

This study has generated direct evidence of the formation of (Ba,Sr)SO_4_ solid solutions with substantial Sr mole fractions in solids precipitated from aqueous solution conditions relevant to industrial waste streams such as FPW. Efficient removal of Sr is key to reducing treatment and disposal costs for industrial wastewaters. In our experiments, the measured final aqueous concentrations of Ba^2+^ (2.1–24.1 mg/L) and Sr^2+^ (3.9–19.4 mg/L) approach the U.S. EPA MCLs for drinking water, indicating that coprecipitation is promising for FPW treatment. Thus, treatment systems employing sulfate precipitation for Ba removal may be able to also achieve Sr removal without adding additional treatment processes.

The initial Ba^2+^ (29.4 and 206 mg/L) and Sr^2+^ (8.3 and 43.8 mg/L) concentrations in our experiments are in the lower range (Ba^2+^ range: 76–13,600 mg/L; Sr^2+^ range 46–5,350 mg/L) of those reported for Marcellus shale FPW (Haluszczak *et al.*, 2013). Many FPW sources will have substantially higher concentrations of both Ba^2+^ and Sr^2+^ that will allow for the formation of pure BaSO_4_, pure SrSO_4_ and (Ba,Sr)SO_4_ solid solutions. In a more concentrated solution, both thermodynamic and kinetic models would predict an increased value of X_SrSO4_.

This study also demonstrates the value of SS-AS theory for predicting trace element incorporation in precipitation treatment systems. For the conditions studied, an endmember solubility model would predict precipitation of only BaSO_4_ and no solid solution. Coprecipitation is likely ubiquitous in wastewater treatment facilities employing any form of chemical precipitation and, given their potential influence on contaminant removal, these reactions should be considered during the design phase.

In the case of barite/celestine precipitation, SS-AS theory predicts that the thermodynamic endpoint can be shifted toward greater Sr in the solid phase by increasing the sulfate dose to increase the value of ΣΠ or by increasing the ratio of Sr^2+^ to Ba^2+^ in solution. While the specific recommendations will vary based on the solubilities of the relevant minerals and the relative ion concentrations in solution, including SS-AS modeling is an important first step in predicting contaminant removal. For example, these models can be used to optimize conditions to achieve aqueous discharge requirements while minimizing sulfate use and sludge generation.

Across all measurements, both nano-XRF and bulk XRF show that in a barite/celestine coprecipitation reaction Sr incorporation is greatly enhanced over SS-AS thermodynamic predictions. This implies the formation of metastable solid solutions. Kinetic factors likely play an important role in coprecipitation reactions in wastewater treatment systems, where initial conditions are always far from equilibrium. The link between increased *SI* and increased precipitation rate, and between increased precipitation rate and increased incorporation of the more soluble compound suggests that *SI* can be a good indicator of contaminant incorporation.

In the barite/celestine case, it appears that accelerating barite precipitation by increasing barite *SI* can be used to produce metastable solids with significantly greater Sr content. In an FPW treatment facility increasing barite *SI* could be accomplished either by adding more Na_2_SO_4_ or by concentrating the wastewater to increase the Ba^2+^ concentration. Applications such as treatment of FPW from oil and gas production and other industrial wastewaters containing multiple metals or metalloids appear to be good candidates for a thermodynamically and kinetically optimized coprecipitation treatment approach.

## Supplementary Material

Supplemental data
