# Supplementary material for: Metals Coprecipitation with Barite: Nano-XRF Observation of Enhanced Strontium Incorporation
Source: Environ Eng Sci. 2020 Apr 6;37(4):235–45. doi: 10.1089/ees.2019.0447 (PMC7175618; doi:10.1089/ees.2019.0447)
Supplement: Supplemental data [file Supp_Data.pdf]

Supplementary Data

Glynn Solution Method: Conservation of Mass and Conservation of Charge

Glynn *et al.* (1990) developed a method to solve for the equilibrium composition of the solid and aqueous phases by introducing additional equations for conservation of mass and conservation of charge in the solid. For the two substituting cations conservation of mass is expressed as:

$$n^0X^0_{BA} + M_{H_2O}m^{T,0}_{B^+} = nX_{BA} + M_{H_2O}m^T_{B^+}. \qquad \text{(S1)}$$

$$n^0X^0_{CA} + M_{H_2O}m^{T,0}_{C^+} = nX_{CA} + M_{H_2O}m^T_{C^+}. \qquad \text{(S2)}$$

where n is the number of moles of solid, M_{H_2O} is the mass of water in the system, m^T_i is the total molality of species i, and

$$\{C^+\}_{eq} = \frac{\gamma_{C^+}K_{CA}\gamma_{CA}(n_0X^0_{CA} + M_{H_2O}m^{T,0}_{C^+})}{K_{CA}\gamma_{CA}M_{H_2O}(\tau+1) + \gamma_{C^+}\{A^-\}\left(n_0 + M_{H_2O}\left(m^{T,0}_{A^-} - (\rho+1)\right)\frac{\{A^-\}}{\gamma_{A^-}}\right)} \qquad \text{(S5)}$$

$$\{B^+\}_{eq} = \frac{\gamma_{B^+}K_{BA}\gamma_{BA}(n_0X^0_{BA} + M_{H_2O}m^{T,0}_{B^+})}{K_{BA}\gamma_{BA}M_{H_2O}(\sigma+1) + \gamma_{B^+}\{A^-\}\left(n_0 + M_{H_2O}\left(m^{T,0}_{A^-} - (\rho+1)\frac{\{A^-\}}{\gamma_{A^-}}\right)\right)} \qquad \text{(S6)}$$

$$\{A^-\}_{eq} = \frac{\gamma A^-}{(\rho+1)}\left[\frac{(\sigma+1)\gamma_{BA}K_{BA}(n_0X^0_{BA} + M_{H_2O}m^{T,0}_{B^+}}{K_{BA}\gamma_{BA}M_{H_2O}(\sigma+1) + \gamma_{B^+}\{A^-\}\left(n_0 + M_{H_2O}\left(m^{T,0}_{A^-} - (\rho+1)\frac{\{A^-\}}{\gamma_{A^-}}\right)\right)} + \frac{(\tau+1)\gamma_{CA}K_{CA}(n_0X^0_{CA} + M_{H_2O}m^{T,0}_{C^+}}{K_{CA}\gamma_{CA}M_{H_2O}(\tau+1) + \gamma_{C^+}\{A^-\}\left(n_0 + M_{H_2O}\left(m^{T,0}_{A^-} - (\rho+1)\frac{\{A^-\}}{\gamma_{A^-}}\right)\right)} + m^{T,0}_{A^-}\left(m^{T,0}_{B^+} + m^{T,0}_{C^+}\right)\right] \qquad \text{(S7)}$$

the superscript 0 denotes the initial value. Total molality accounts for multiple aqueous species using a speciation factor [Supplementary Data Eq. (S4)]. Throughout this work, the geochemical model PHREEQC (Parkhurst and Appelo, 2013) with the Pitzer model was used to calculate aqueous-phase activity coefficients and speciation factors. An equation expressing conservation of charge in the solid is also required:

$$m^T_{A^-} - m^{T,0}_{A^-} = m^T_{B^+} - m^{T,0}_{B^+} + m^T_{C^+} - m^{T,0}_{C^+}. \qquad \text{(S3)}$$

Equations (S1)–(S3) are combined with the mass action equations and rearranged in terms of the equilibrium activities [Supplementary Data Eqs. (S5–S7)].

Relationship for Total Molality

The relationship for total molality for solute i is

$$m^T_i = \frac{\{i\}}{\gamma_i}(\tau+1). \qquad \text{(S4)}$$

where $\{i\}$ is the ion activity in the aqueous phase. γ_i is the aqueous-phase activity coefficient and τ is a speciation factor to account for the ions that are present in complexed form. In the following, ρ is the speciation factor associated with the anion A^-, σ is the speciation factor for B^+, and τ is the speciation factor for C^+.

Equations for Equilibrium Activities

The equations for aqueous-phase activities for solutes in equilibrium with solid solution are derived by combining the equations for conservation of mass and conservation of charge [Eqs. (S1)–(S3)] with mass action equations [Eqs. (3) and (4) in the main manuscript] after Glynn *et al.* (1990). Values of n and X_{BA} are then found using Equations S1 and S2 and considering that $X_{BA} + X_{CA} = 1$.

Equations for Reaction Path

The reaction path describing the change in the aqueous-phase composition is calculated after solving for the equilibrium composition (so that $\{A^-\}_{eq}$, $\{B^+\}_{eq}$, $\{C^+\}_{eq}$, X_{BA}, and X_{CA} are known). These equations are derived by combining Equations (11), (12), and (14) and the relationship between total molality and aqueous activity [Eq. (S1)]. The reaction path is represented by all the initial activities of A^-, B^+, and C^+ that would result in the same equilibrium activities and is found by varying the solid-to-water ratio ($\frac{n}{M_{H_2O}}$) between its initial value before reaction and its equilibrium value in the following equations:

$$\{A^-\}_i = \frac{n}{M_{H_2O}}\gamma_{A^-} + (\rho+1)\{A^-\}_{eq} \qquad \text{(S8)}$$

$$\{B^+\}_i = \frac{n}{M_{H_2O}}\gamma_{B^+}X_{BA} + (\sigma+1)\{B^+\}_{eq} \qquad \text{(S9)}$$

Supplementary Table S1. Initial Solution Conditions for the Hypothetical Reaction Paths in FIGURE 1

	$[Ba^{2+}]$ (mM)	$[SO_4^{2-}]$ (mM)	$[Sr^{2+}]$ (mM)	$X_{Ba(aq)}$	$\log \Sigma\Pi$
A	0.9	1.8	0.1	0.92	−5.74
B	0.055	0.055	0.02	0.74	−8.38
C	0.165	0.18	0.06	0.74	−7.39
D	1.2	1.2	0.5	0.74	−5.69
E	0.5	2	0.6	0.52	−5.66
F	1	1	1	0.53	−5.70

$$\{C^+\}_i = \frac{n}{M_{H_2O}}\gamma_{C^+}X_{CA} + (\tau+1)\{C^+\}_{eq} \tag{S10}$$

Endmember Solubility Model

An example of a endmember solubility model of precipitation of multiple minerals with a common anion is the standard precipitation model implemented in PHREEQC (Parkhurst and Appelo, 2013). Such a model predicts zero $SrSO_4$ precipitation in both the high and low *SI* conditions studied in this work. This is because of the assumption that no solid solutions form and precipitation occurs sequentially, beginning with the mineral that has the highest *SI*. Solutes associated with that mineral would be removed from solution until its *SI* equals zero. The *SI* values of all minerals are then recalculated based on the new aqueous ion concentrations. If any minerals remain supersaturated, the mineral with the highest *SI* is then precipitated. For the high *SI* condition, this model leads to the prediction of no $SrSO_4$ precipitation sulfate even though the solution is initially supersaturated with respect to $SrSO_4$. The initial SI_{BaSO4} is 3.80 and the initial SI_{SrSO4} is 0.11, so barite is precipitated first. Barite precipitation reduces the SO_4^{2-} concentration from 1.5 mM to 1.6×10^{-2} mM and the resulting solution is undersaturated in celestine (SI_{SrSO4} is −1.70) so that no celestine is predicted to precipitate. Sulfate is the limiting ion in this case. If the initial sulfate concentration were doubled, there would be enough sulfate remaining after barite precipitation to allow celestine precipitation as well.

Nano-XRF Imaging and Elemental Quantification

Nano-XRF imaging of individual particles at the HXN Beamline at NSLS-II was conducted using Multilayer Laue Lenses with nanofocusing optics, which resulted in a 12 × 13 nm focused beam. A three-element silicon drift detector (SDD, Vortex), positioned perpendicular to the X-ray beam, was used for collecting XRF spectra. A pixel array detector (512 × 512 pixels and 55 μm/pixel, Merlin), positioned at 0.5 m downstream of the sample, was used for phase-contrast imaging, which was used to map phase density and constrain particle thickness. For XRF imaging, raster scanning was used by continuous fly-scanning across individual barite particles at 30–60 nm steps with dwell times from 100 to 250 ms. XRF spectra were collected at 16.118 keV to maximize sensitivity to Sr.

Spectral fitting of XRF data was performed using PyXRF (Li *et al.*, 2017) to quantify emission intensities and create maps of S Kα, Ba Lα, and Sr Kα. Analysis that leveraged NIST NRLXRF software (Birks *et al.*, 1977) was used to interpret emission intensities (counts per second), and ultimately compute Ba and Sr mole fractions. A calibration curve was generated for an array of barite and celestine solid solution stoichiometries for amounts of Sr ranging from no substitution to complete substitution. The theoretical fluorescence intensities of the Ba Lα and Sr Kα emission lines were computed using NRLXRF. The ratios of emission intensities of Ba Lα to Sr Kα were used to compute X_{SrSO_4}. Particle thickness was estimated based on particle aspect ratios in SEM images. NRLXRF, which accounts for X-ray fluorescence escape depth, demonstrated the relative insensitivity of the quantitative XRF results to uncertainty in sample thickness.

Supplementary References

Birks, L.S., Gilfrich, J.V., and Criss, J.W. (1977). *NRLXRF, A Fortran Program for X-Ray Fluorescence Analysis: Users' Guide*. Washington, D.C.: Naval Research Laboratory.

Glynn, P.D., Eric, J.R, Plummer, L.N., and Eurybiades, B. (1990). Reaction Paths and Equilibrium End-Points in Solid-Solution Aqueous-Solutions Systems. *Geochim. Cosmochim. Acta* 54, 267.

Li, L., Hanfei, Y., Wei, X., Dantong, Y., Annie, H., Wah-Keat, L., Li, L., Stuart, C., and Yong, C. (2017). PyXRF: Python-Based X-Ray Fluorescence Analysis Package. In A. Somogyi and B. Lai, Eds., *X-Ray Nanoimaging: Instruments and Methods III* 30. San Diego, CA: SPIE.

Parkhurst, D.L., and Appelo, C.A.J. (2013). Description of Input and Examples for PHREEQC Version 3-A Computer Program for Speciation, Batch-Reaction, One-Dimensional Transport, and Inverse Geochemical Calculations. In *U.S. Geological Survey Techniques and Methods, Book 6*, 497 p. https://doi.org/available only at http://pubs.usgs.gov/tm/06/a43.
